# Quantitative analysis of shoulder function and strength after breast reconstruction: A retrospective cohort study

**DOI:** 10.1097/MD.0000000000010979

**Published:** 2018-06-15

**Authors:** Yujin Myung, Bomi Choi, Heeyeon Kwon, Chan Yeong Heo, Eun-Kyu Kim, Eunyoung Kang, Jae Hoon Jeong, Eun Joo Yang

**Affiliations:** a Department of Plastic and Reconstructive Surgery; b Department of Surgery; c Department of Rehabilitation Medicine, Seoul National University Bundang Hospital, Seongnam, Republic of Korea.

**Keywords:** breast cancer, breast reconstruction, muscle strength, rehabilitation, shoulder

## Abstract

Immediate breast reconstruction (IBR) after mastectomy has been proven to affect patient quality of life, psychological well-being, and functional capacities. In the present study, we aimed to investigate the effect of breast reconstruction on postoperative shoulder function and muscle performance by evaluating isokinetic muscle performance tests.

A retrospective chart review to collect data on shoulder function analysis with isokinetic muscle performance testing was performed among patients who received mastectomy with IBR from July 2013 to March 2015. Patients were categorized into 4 groups: a control group that underwent mastectomy without reconstruction, a tissue expander/implant insertion group, a pedicled latissimus dorsi (LD) flap group, and a free transverse rectus abdominis muscle (TRAM) flap group.

Analysis of the groups at 1 to 3, 4 to 6, 7 to 9, 10 to 12, and 13 to 15 months postoperatively showed significant shoulder function improvement in the tissue expander/implant and TRAM groups as measured by linear regression analysis. Compared with the control group, patients who received immediate reconstruction with tissue expander/implant insertion or a TRAM flap showed statistically significant improvement in shoulder function after mastectomy.

IBR with a TRAM flap or tissue expander/implant insertion were more beneficial for shoulder rehabilitation and for regaining function compared to mastectomy alone and breast reconstruction with a LD flap.

## 1 Introduction

With an increasing number of breast cancer survivors, reducing the morbidity of treatment-side-effects has become the focus of clinical trials and research in recent years. Despite the widespread acceptance of breast conservation as a therapy for early stage breast cancer, recent reports indicate that mastectomy rates have begun to rise.^[1,2]^ The factors that influence these treatment decisions involve issues regarding access to health care, concerns for cancer recurrence, and the impact of surgery on body image and sexuality.^[3]^ Immediate breast reconstruction (IBR) has been shown to improve the quality of life of breast cancer patients by offering emotional and social benefits while providing oncological safety.^[4,5]^ The percentage of patients undergoing breast reconstruction is steadily increasing.^[1]^

Although implant-based procedures are currently one of the most common surgeries performed in the reconstructive surgical field,^[2,6,7]^ the number of flap-based options has increased dramatically in recent years. Comparing risks across procedure types and weighing the relative risks and benefits of each operation can prove challenging.^[8]^ It is well known that postoperative upper limb disability negatively affects physical function and leads to a poor quality of life in breast cancer survivors.^[9]^ However, among shoulder morbidity studies in breast cancer patients, studies based on differences in reconstruction techniques are rare.^[10]^ For patients and their providers to make truly informed reconstruction decisions, comparative research is needed to assess the risks of these procedures such as shoulder morbidity.

Previous studies reported shoulder function deficits after breast reconstruction with the latissimus dorsi (LD) flap,^[11,12]^ the transverse rectus abdominis myocutaneous (TRAM) flap,^[13]^ and with a subpectoral prosthetic reconstruction.^[14]^ Button et al^[15]^ noted decreased shoulder function after flap surgery, although Lee et al^[13]^ reported that IBR has advantages for preserving scapular resting alignment but did not provide benefits for shoulder morbidity on short-term follow-up. Many studies have been based on subjective self-reporting that might have been affected by psychological factors.^[15]^ A lack of objective studies assessing shoulder strength after different reconstruction procedure has lead to difficulties in decision making regarding the optimal reconstruction plan after breast surgery. To evaluate the effect of the various methods of IBR on shoulder function, we compared shoulder muscle strength using isokinetic testing in patients who underwent IBR.

## 2 Patients and methods

### 2.1 Participant selection

**Figure 1:**
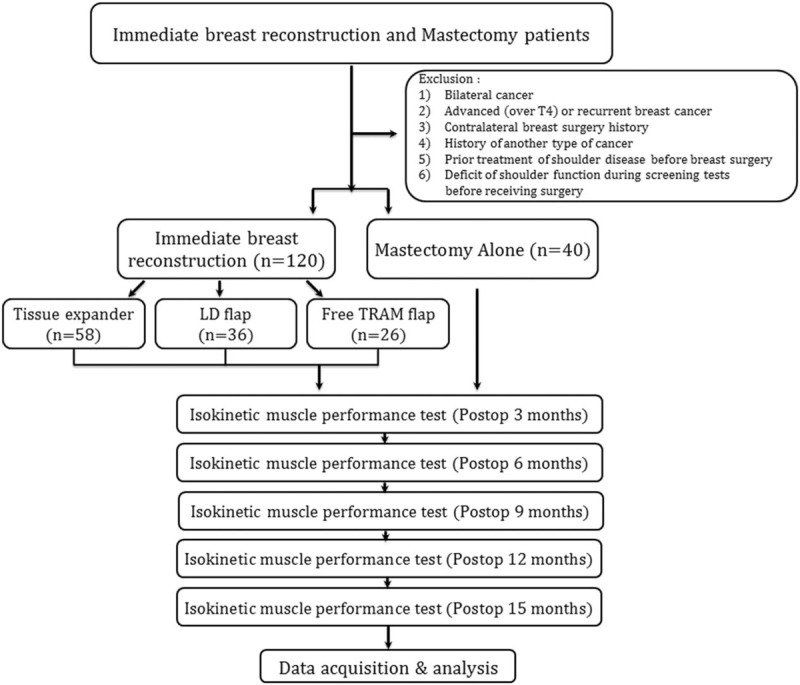
Flow sheet of patient recruitment and study design. LD = latissimus dorsi, TRAM = transverse rectus abdominis musculocutaneous.

The study followed the guidelines of World Medical Association Declaration of Helsinki, and received permission from the institutional review board of Seoul National University Bundang Hospital. A retrospective chart review was performed to collect the shoulder function measurements of patients who underwent IBR from July 2013 through May 2015 (Fig. 1). The IBR groups included the prosthetic reconstruction group, the LD flap group, and the TRAM flap group. The method of reconstruction was selected according to routine clinical care. Data were also collected for a control group that underwent mastectomy alone without reconstruction during the same time period. Baseline characteristics of patients from both the immediate reconstruction and mastectomy alone groups were matched with regard to pathology and mastectomy types, status of neoadjuvant or adjuvant chemotherapy, radiotherapy and hormone therapy (Table 1). Exclusion criteria included bilateral or advanced cancer over stage T4 or recurrent breast cancer, contralateral breast surgery for benign disease, a history of another type of cancer, and prior treatment of a shoulder disease before breast surgery. In addition, all patients received screening tests performed by physical therapist before the admission, and the patients with confirmed deficit of shoulder function over 30% compared to average norm values were excluded from the study. Total sample size was calculated and was based on the power of 85%. The target sample size was 30 participants in each group. Patients who underwent mastectomy were selected and matched for the control group. In matching the groups, we cautiously considered factors that might affect shoulder function, axillary lymph node dissection and radiation therapy, which were known as important factors for shoulder disability, were matched. Other factors such as age, body mass index (BMI), and cancer stage were also matched. The patients’ postoperative shoulder function was evaluated with isokinetic muscle performance test (IMPT) measured during postoperative hospital rehabilitation visits within 1 to 15 months after the operation. The authors confirmed the study reports with STROBE checklist for cohort studies.

**Table 1:**
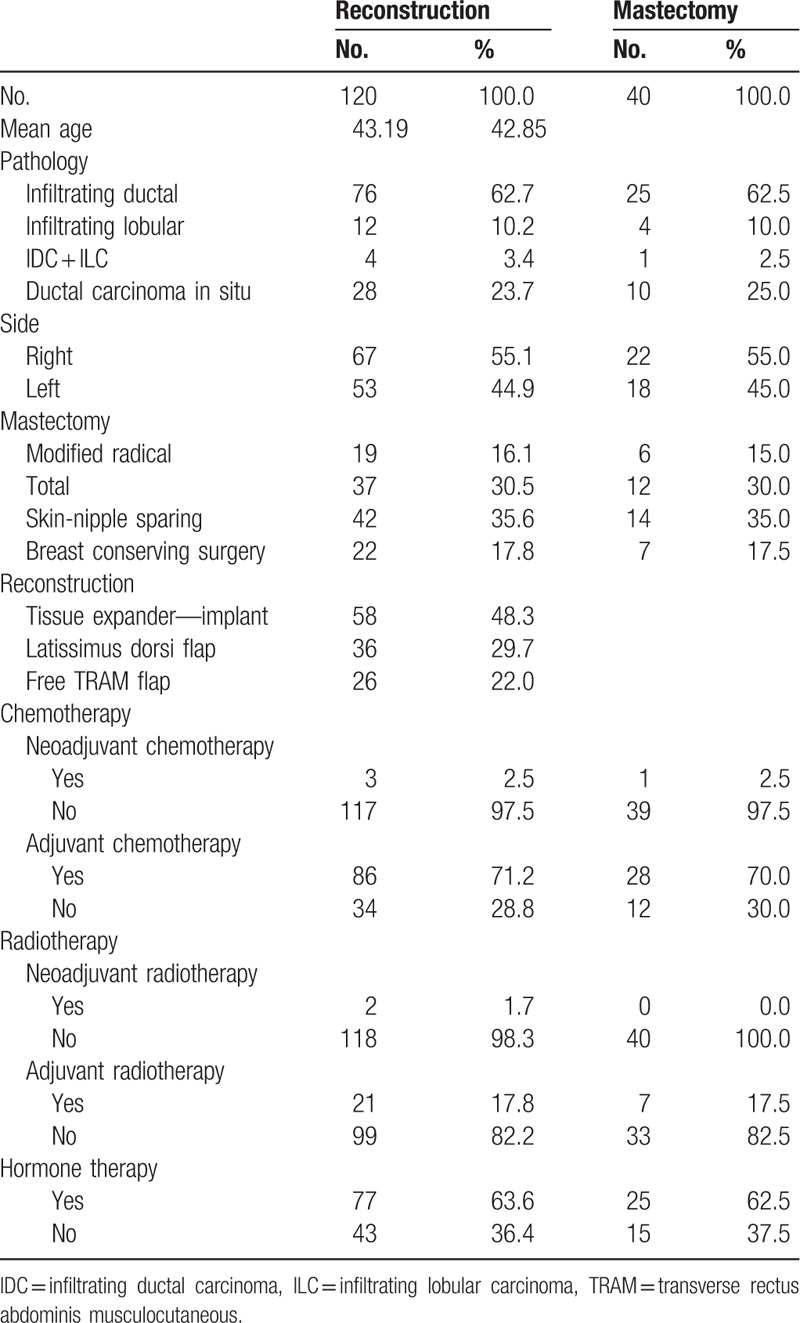
Patient characteristics.

### 2.2 Surgical options

Surgical options included tissue expander/implant insertion, LD flap, and TRAM flap. The selection of the reconstruction method performed after mastectomy or wide local excision with breast conserving surgery was made by both the surgeon and the patient after a detailed and thorough discussion and consultation regarding the risks and benefits of the various surgical reconstruction methods.

### 2.3 Tissue expander/implant insertion

After mastectomy was performed by the general surgeon, reconstruction with expander insertion was performed by a reconstructive surgeon using the same incision that was previously made during the mastectomy. First, the inferior portion of the costal origin of the pectoralis major muscle was completely detached, and the medial portion of the sternal origin was conserved. The amount of detachment of the upper portion of the pectoralis major was controlled to maintain symmetry with the opposite breast. After complete detachment of the lower portion of the pectoralis muscle, an acellular dermal matrix was applied and anchoring sutures were placed in order to reconstruct a new inframammary fold on the lower part of the breast. Initial insertion of the expander was followed by 1 to 3 months of tissue expansion and a consolidation period of at least 3 months. A final operation to remove the expanders and insert permanent silicone implants was then performed. The shape and size of the implant was selected to match maximal symmetry with the opposite breast.

### 2.4 Latissimus dorsi flap

In patients who received breast conserving surgery, or in patients who required little volume replacement despite mastectomy, breast reconstruction was performed with a LD muscle or musculocutaneous flap. The lower and medial portion of the LD origin and insertion into the humerus were detached to rotate the muscle freely, without damaging the main pedicle, thoracodorsal artery and vein, or thoracodorsal nerve. If a skin pedicle was needed, the flap was elevated as musculocutaneous flap. In all cases, the donor site was closed primarily.

### 2.5 Transverse rectus abdominis musculocutaneous flap

Following mastectomy, a free abdominal flap was elevated using a muscle sparing transverse rectus abdominis musculocutaneous (TRAM) flap method or a deep inferior epigastric artery perforator flap method. Anastomoses were performed to the internal mammary or thoracodorsal arteries and veins. When using the thoracodorsal vessels as recipients, long thoracic and intercostobrachial nerves were preserved without damage. To achieve a working field for microsurgical anastomosis when using the internal mammary vessels, the pectoralis major muscle between the rib cartilages was ligated, creating a window of approximately 3 cm × 4 cm. To avoid any unnecessary pressure over the vessels, the ligated muscle was not repaired after completing the anastomosis.

### 2.6 Measurement of shoulder muscle strength

**Figure 2:**
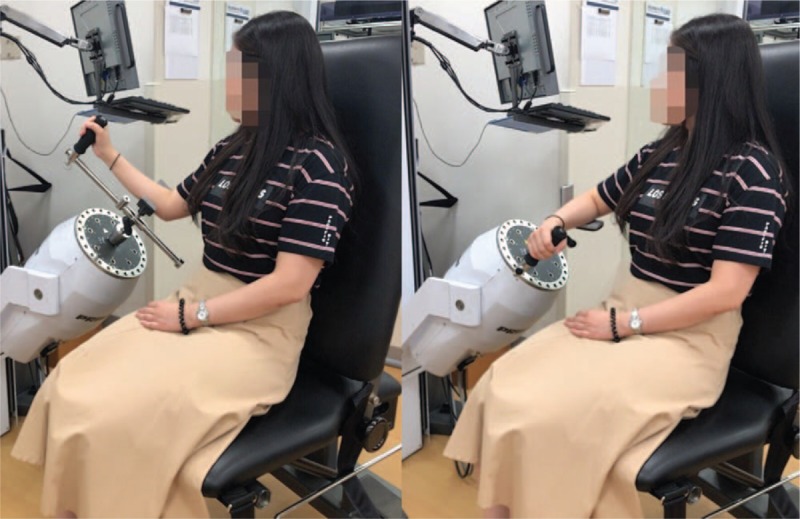
Patient performing isokinetic performance test in outpatient clinic. The patients performed 2 sets of 5 repetitions of isokinetic abduction/adduction (left) and internal rotation/external rotation (right) with a 15-second rest between sets.

Objective measurements of shoulder muscle function using the Biodex isokinetic machine (Biodex System 3 Dynamometer; Biodex Medical Systems, Inc., Shirley, NY) were obtained. Participants were asked to perform 2 sets of 5 repetitions of isokinetic abduction/adduction and internal rotation/external rotation with a 15-second rest between sets (Fig. 2). Participants were asked to perform the test by exerting maximum pressure on the isokinetic arm throughout the entire range of movement. The total duration of the test for each side was approximately 1 minute. Peak torques of abduction, adduction, and external and internal rotation were measured and recorded. Additionally, the total work (J) obtained throughout the test was recorded for shoulders on both the treated and untreated sides. Measurements of shoulder function on the reconstructed breast side were designated as the experimental group and those of the contralateral side as the control group, and the amount of deficit in percentage was obtained in each patient.

### 2.7 Data collection

Information including patient age, height, body weight, BMI, previous operation history, and medical history were collected. Additionally, information regarding the use of neoadjuvant/adjuvant chemotherapy and postoperative radiotherapy was collected. For the comparison between patients with breast reconstruction and mastectomy alone, a total of 40 breast cancer patients who received mastectomy without IBR that were propensity matched for age, pathology, mastectomy type, chemotherapy, radiotherapy, and hormone therapy with the reconstruction cohort were additionally included in the retrospective review of IMPT measurements. The shoulder function data of the mastectomy alone group were compared with those of the groups undergoing the various breast reconstruction procedures.

### 2.8 Statistical analysis

First, intragroup analyses of the expander group, the LD flap group, and the TRAM flap group were performed, assessing the percentage of deficit at each of the IMPT measurement time points for abduction and adduction peak torque. IMPT results were analyzed based on the time since surgery: 3, 6, 9, 12, and 15 months. The measured data were compared with the linear regression method, and for results that showed statistical significance, a post hoc Bonferroni test was performed.

To analyze the differences between the effects of the various reconstruction methods on postoperative shoulder function, we performed an intergroup comparison of the IMPT results using the one-way ANOVA method with post hoc test and investigated whether the difference was statistically significant. In addition, the IMPT results were assessed and analyzed using 2 distinctive factors, surgery method and postoperative time interval, with a multivariate generalized linear model. The same methods were used to compare the results with the propensity-matched control mastectomy group. Statistical significance was set at *P* < .05 in every analysis, and all data management and statistical analyses were performed with SPSS version 21.0 (IBM Corporation, Armonk, NY).

## 3 Results

### 3.1 Patient characteristics

A total of 120 patients who underwent IBR were recruited for our retrospective analysis of shoulder function after surgery. Fifty-eight patients underwent a tissue expander/implant based reconstruction, 36 patients underwent a LD flap reconstruction, and 26 patients underwent a free TRAM flap reconstruction. Forty additional patients who underwent only mastectomy were added for retrospective review and IMPT score comparison with each of the reconstruction groups. The median patient age was 43 years in the reconstruction groups and 42 years in the mastectomy only group (Table 1).

### 3.2 Strength profile difference vs postoperative duration

**Figure 3:**
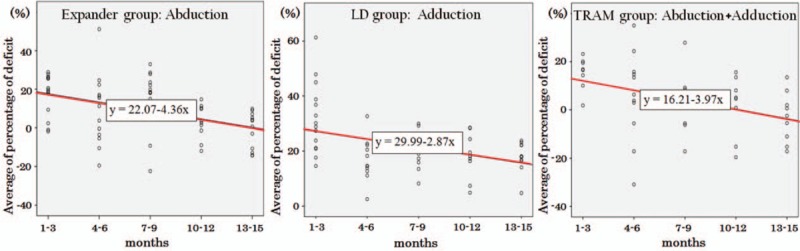
Graphic representation of linear regression analysis regarding percentage deficit. Abduction peak torque in tissue expander/implant insertion group (left). Adduction peak torque in latissimus dorsi pedicled flap group (middle). Abduction and adduction total work in the transverse rectus abdominis musculocutaneous flap group (right). LD = latissimus dorsi, TRAM = transverse rectus abdominis musculocutaneous.

In the intragroup comparison of each of the reconstruction groups, the tendency for improvement in shoulder function based on the length of the postoperative period was analyzed using linear regression analysis. Differences between the 5 different IMPT testing time intervals (postoperative 3, 6, 9, 12, and 15 months) were investigated, and the trend toward improvement in shoulder function was analyzed with simple linear regression analysis (Table 2). In every reconstruction group, including the expander/implant, LD flap, and TRAM flap group, the operated side's deficit percentage in adduction peak torque significantly decreased with an increasing length of the postoperative duration. In the expander/implant group, the deficit percentage of abduction peak torque also showed a significant decrease over time, with eventual normalization of shoulder function. Comparing the *R*^2^ value, the TRAM flap group showed the largest gain in the operated side shoulder function during the postoperative period (Fig. 3). In all 3 groups, the external and internal rotation torque also showed a decrease in deficit percentage over time; however, these differences were not statistically significant.

**Table 2:**
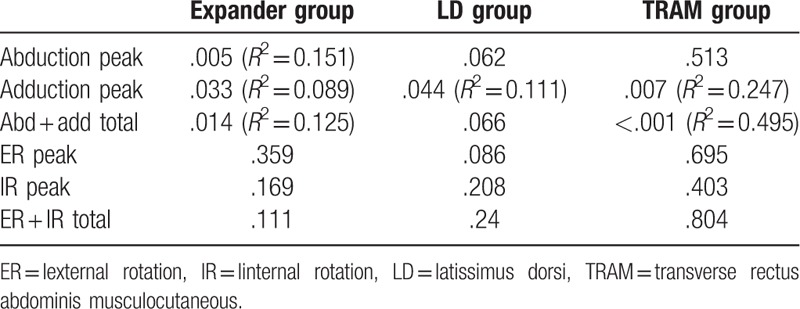
Linear regression analysis *p* and *R*^2^ values.

### 3.3 Strength profile difference between the surgical procedures

IMPT measurements at 3 months postoperatively did not show a significant difference between the reconstruction procedures with regard to operated-side shoulder movement and function. However, at 6 months postoperatively, the LD flap group had a more significant functional deficit of the operated-side shoulder in peak torque of adduction and internal rotation, compared with the TRAM or expander/implant groups. The shoulder function deficit tended to improve in the LD flap patients at the 9-, 12-, and 15-month postoperative IMPT tests. In addition, we analyzed the relationship between the reconstruction methods and shoulder function improvement using a generalized linear model. The TRAM flap group showed the most rapid and significant functional improvement in abduction and adduction peak torque, followed by the expander/implant and LD flap groups. Internal rotation peak torque showed similar results. In contrast, external rotation was not significantly different among the different reconstruction groups (Table 3).

**Table 3:**
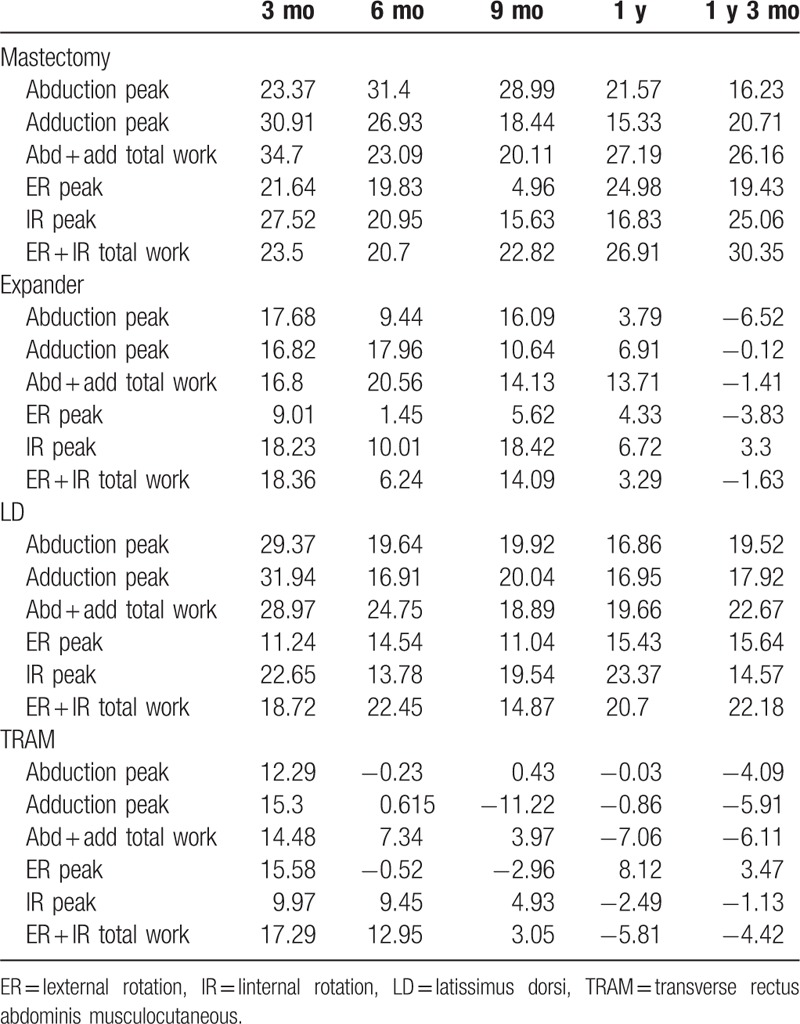
Percentage deficit compared to contralateral shoulder.

### 3.4 IMPT profile comparison between mastectomy alone and immediate reconstruction

**Figure 4:**
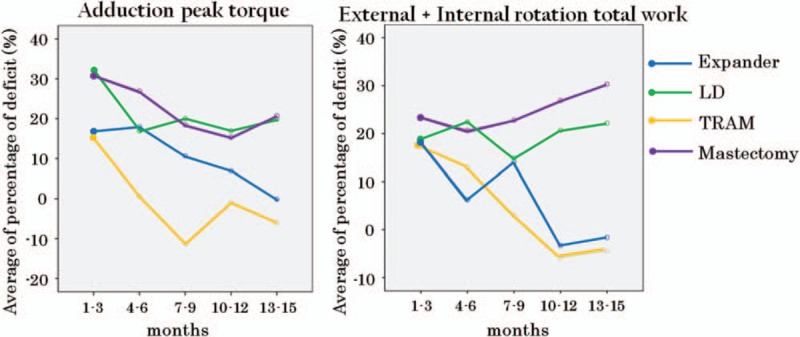
Comparison of percentage deficit in adduction peak torque (left) and external + internal rotation total work (right). Generalized linear model analysis showed statistically significant difference of the 10- to 12 and 13- to 15-month results in both measurements. LD = latissimus dorsi, TRAM = transverse rectus abdominis musculocutaneous.

IMPT results of mastectomy patients who did not receive immediate reconstruction were not significantly different during the postoperative period compared with those of the LD flap reconstruction group. However, shoulder function improvement in the expander/implant and TRAM groups showed significant improvement compared with that seen in mastectomy only patients with regard to abduction/adduction and external/internal rotation peak torques (Fig. 4).

## 4 Discussion

Recently, more attention has been given to shoulder rehabilitation with a focus on muscle strengthening and range of motion maintenance in breast cancer patients after mastectomy and breast reconstruction.^[16]^ However, to our knowledge, ours is the first study to objectively measure postoperative shoulder function after various surgical methods of breast reconstruction. Strength and range of motion of the shoulder joint are critical for a better postoperative quality of life in cancer survivors. The findings of the present study provide valuable information regarding postoperative quality of life and physical function and provide guidance for discussing treatment plans and options with breast cancer patients.

Quantitative analysis of muscle function with isokinetic testing after LD flap breast reconstruction was previously reported by Forthomme et al^[17]^ and van Huizum et al.^[18]^ Forthomme performed a retrospective analysis of preoperative and postoperative muscle power in 20 patients, and reported that abduction, adduction, and internal rotation of the operated-side's shoulder were all significantly decreased after an LD flap operation. van Huizum also reported that 12 patients who underwent an LD flap breast reconstruction showed a 19% (8.8 Nm) decrease in shoulder strength torque compared with a control group; however, no significant difference was noted on comparison between the operated and nonoperated sides. Both studies were limited by their small size and uncontrolled timing of muscle function measurements that may have led to significant selection bias compared with the present study.

In the majority of studies that have assessed shoulder function and quality of life after an LD flap breast reconstruction, a disability of the arm, shoulder, and hand (DASH) questionnaire was used as an evaluation tool.^[18–20]^ In a systematic review published in 2014, Lee and Mun^[21]^ reported that based on seven previous articles employing DASH questionnaire results, patients who received an LD muscle flap breast reconstruction experienced little difficulty with daily activities during the postoperative period; however, there was significant discomfort when performing sports or art activities.

In contrast, there have been limited reports regarding postoperative shoulder function after breast reconstruction methods such as tissue expander/implant insertion or abdominal free flap reconstruction. In 2011, Harrington et al^[20]^ reported a limitation of shoulder movement and a strength decrease in breast cancer survivors compared with healthy individuals, based on DASH scores, Penn shoulder scores, range of motion, and hand held dynamometer measurements. In a systematic review reported by Hidding et al^[22]^ in 2014, patients who received IBR showed less impairment of upper extremity function compared with those who underwent only mastectomy. However, variables such as the methods of reconstruction or mastectomy, axillary lymph node dissection, and radiotherapy were not included in the analysis.

The results of the present study show that IBR with tissue expansion or a free TRAM flap had positive effects on postoperative shoulder function and recovery compared with mastectomy alone or LD flap reconstruction. There are a number of possible reasons for these results. First, unlike LD flap reconstruction, implant-based reconstruction and TRAM flap reconstruction do not affect the muscle strength or power of the shoulder, and thus allow a more efficient response to postoperative physiotherapy, resulting in a more rapid functional recovery. TRAM surgery also has no effect on the muscles of the upper extremity, including the shoulder, except for donor-related morbidity that affects the integrity of the rectus abdominis muscle and the abdominal fascia. If the internal mammary artery and vein are used as recipient vessels, a small muscular window is created on the sternal origin side, but most of the muscle is left undisturbed. In tissue expander/implant reconstruction, the thoracic origin and portions of the sternal origin of the lower pole of the chest are separated, but the isolated muscle portions are reconnected to the chest wall using an acellular dermal matrix to maintain muscle continuity. On the other hand, the use of a pedicled LD flap involves detachment of both the origin and insertion of the LD muscle, and some reduction in postoperative shoulder function is expected.

Postsurgical pain related to skin defects and contractures, as well as depression of the shoulder girdle from shortening of the pectoralis major and minor muscles, can lead to protective posturing in patients undergoing mastectomy without IBR. Additionally, as reported in numerous previous studies, IBR has a positive impact on the patient's self-image and overall psychological well-being, and is related to better patient compliance and cooperation with postoperative rehabilitation therapy.

It has been shown that the survival and recurrence rates after breast conserving surgery are almost identical to those of patients undergoing total mastectomy and radiation therapy, and that the choice of treatment should be made after the patient is fully informed of the risks and benefits of each procedure by the surgeon. In this study, tissue expander/implant or TRAM after mastectomy provided an improved prognosis with regard to shoulder function recovery than did mastectomy or breast conserving surgery followed by an LD flap reconstruction. This is important information for patients and surgeons when choosing the most appropriate option for breast cancer surgery.

The present study is limited by a possible selection bias due to the retrospective nature of the study. Not all study patients underwent IMPT testing during each postoperative time period. Additionally, mastectomy patients were included in the control group. However, as can be seen from the above results, shoulder function tended to improve over time, and patients who were not compliant with the follow-up schedule and did not have IMPT tests likely had good functional recovery and did not need to visit the clinic again. Therefore, this would not have had a significant impact on the outcome of the entire study. Second, there were sample size differences between groups. If the sample size differs between groups, the statistical power decreases compared to that in case of groups of equal size. However, there is no statistical contraindication for performing parametric tests when comparing groups with different sample sizes. We performed an intergroup comparison using not only the one-way ANOVA method, but also the post hoc test, and confirmed that the comparison of groups was meaningful, although the sizes of the groups were largely variable. This should be taken into consideration when interpreting our results. Third, the impact of postoperative radiotherapy could not be assessed in this study. Although postoperative radiotherapy is a mainstay of treatment after breast conserving surgery, a separate comparison study between breast conserving surgery patients and breast conserving surgery patients undergoing an LD flap was not included owing to the small number of patients; we are currently gathering additional information and plan to report additional findings in the near future. Fourth, the IMPT tests were carried out every 3 months as per routine clinical care protocol. It was based on the clinical decision of the rehabilitation specialists. However, it is difficult to conclude that the 3-month interval is appropriate based on the results of this study. We feel that further studies are required to investigate the appropriate test intervals.

In conclusion, the present study assessed the relationship between various breast reconstruction methods, including tissue expander/implant, TRAM-free flap, and LD-pedicled flap, and patient postoperative shoulder function recovery. We found that recovery and rehabilitation of postoperative shoulder function was significantly better in patients undergoing immediate reconstruction than in those without reconstruction, and among various reconstruction methods, TRAM-free flap and tissue expander/implant showed better postoperative function than the LD-pedicled flap. This study provides valuable information regarding the optimal surgical treatment of patients with breast cancer.

## Author contributions

**Conceptualization:** Yujin Myung, Chan Yeong Heo, Eun-Kyu Kim, Eunyoung Kang, Jae Hoon Jeong, Eun Joo Yang.

**Data curation:** Yujin Myung, Bomi Choi, Heeyeon Kwon, Chan Yeong Heo, Eun-Kyu Kim, Eunyoung Kang, Jae Hoon Jeong, Eun Joo Yang.

**Formal analysis:** Yujin Myung, Bomi Choi, Eun Joo Yang.

**Methodology:** Yujin Myung, Bomi Choi, Eun-Kyu Kim, Eunyoung Kang, Jae Hoon Jeong.

**Software:** Heeyeon Kwon.

**Supervision:** Chan Yeong Heo, Eun-Kyu Kim, Eunyoung Kang, Eun Joo Yang.

**Visualization:** Heeyeon Kwon.

**Writing – original draft:** Yujin Myung, Jae Hoon Jeong, Eun Joo Yang.

**Writing – review & editing:** Chan Yeong Heo, Eun-Kyu Kim, Eunyoung Kang.

## References

[1] Kummerow KL Du L Penson DF Nationwide trends in mastectomy for early-stage breast cancer. JAMA Surg 2015;150:9–16. 25408966 10.1001/jamasurg.2014.2895

[2] Son BH Kwak BS Kim JK Changing patterns in the clinical characteristics of Korean patients with breast cancer during the last 15 years. Arch Surg 2006;141:155–60. 16490892 10.1001/archsurg.141.2.155

[3] Bellavance EC Kesmodel SB Decision-making in the surgical treatment of breast cancer: factors influencing women's choices for mastectomy and breast conserving surgery. Front Oncol 2016;6:74. 27066455 10.3389/fonc.2016.00074 PMC4810034

[4] Nedumpara T Jonker L Williams MR Impact of immediate breast reconstruction on breast cancer recurrence and survival. Breast 2011;20:437–43. 21601458 10.1016/j.breast.2011.04.006

[5] Heneghan HM Prichard RS Lyons R Quality of life after immediate breast reconstruction and skin-sparing mastectomy—a comparison with patients undergoing breast conserving surgery. Eur J Surg Oncol 2011;37:937–43. 21899982 10.1016/j.ejso.2011.08.126

[6] Losken A Carlson GW Bostwick J III Trends in unilateral breast reconstruction and management of the contralateral breast: the Emory experience. Plast Reconstr Surg 2002;110:89–97. 12087236 10.1097/00006534-200207000-00016

[7] Albornoz CR Cordeiro PG Mehrara BJ Economic implications of recent trends in U.S. immediate autologous breast reconstruction. Plast Reconstr Surg 2014;133:463–70. 24572839 10.1097/PRS.0000000000000039

[8] Wilkins EG Hamill JB Kim HM Complications in postmastectomy breast reconstruction: one-year outcomes of the Mastectomy Reconstruction Outcomes Consortium (MROC) study. Ann Surg 2018;267:164–70. 27906762 10.1097/SLA.0000000000002033 PMC5904787

[9] Nesvold IL Reinertsen KV Fossa SD The relation between arm/shoulder problems and quality of life in breast cancer survivors: a cross-sectional and longitudinal study. J Cancer Surviv 2011;5:62–72. 20972640 10.1007/s11764-010-0156-4 PMC3040353

[10] Freitas-Silva R Conde DM de Freitas-Junior R Comparison of quality of life, satisfaction with surgery and shoulder-arm morbidity in breast cancer survivors submitted to breast-conserving therapy or mastectomy followed by immediate breast reconstruction. Clinics (Sao Paulo) 2010;65:781–7. 20835555 10.1590/S1807-59322010000800009 PMC2933125

[11] Eyjolfsdottir H Haraldsdottir B Ragnarsdottir M A prospective analysis on functional outcomes following extended latissimus dorsi flap breast reconstruction. Scand J Surg 2017;106:152–7. 27369738 10.1177/1457496916655500

[12] Yang JD Huh JS Min YS Physical and functional ability recovery patterns and quality of life after immediate autologous latissimus dorsi breast reconstruction: a 1-year prospective observational study. Plast Reconstr Surg 2015;136:1146–54. 26267396 10.1097/PRS.0000000000001769

[13] Lee BG Kim JK Choi SH Is immediate reconstruction after mastectomy in breast cancer patients beneficial to shoulder function? ANZ J Surg 2018;88:363–8. 27582412 10.1111/ans.13735

[14] Hage JJ van der Heeden JF Lankhorst KM Impact of combined skin sparing mastectomy and immediate subpectoral prosthetic reconstruction on the pectoralis major muscle function: a preoperative and postoperative comparative study. Ann Plast Surg 2014;72:631–7. 23486118 10.1097/SAP.0b013e318269e4ee

[15] Button J Scott J Taghizadeh R Shoulder function following autologous latissimus dorsi breast reconstruction. A prospective three year observational study comparing quilting and non-quilting donor site techniques. J Plast Reconstr Aesthet Surg 2010;63:1505–12. 19819774 10.1016/j.bjps.2009.08.017

[16] Yu SC Kleiber GM Song DH An algorithmic approach to total breast reconstruction with free tissue transfer. Arch Plast Surg 2013;40:173–80. 23730589 10.5999/aps.2013.40.3.173 PMC3665857

[17] Forthomme B Heymans O Jacquemin D Shoulder function after latissimus dorsi transfer in breast reconstruction. Clin Physiol Funct Imaging 2010;30:406–12. 20633032 10.1111/j.1475-097X.2010.00956.x

[18] van Huizum MA Hoornweg MJ de Ruiter N Effect of latissimus dorsi flap breast reconstruction on the strength profile of the upper extremity. J Plast Surg Hand Surg 2016;50:202–7. 27046453 10.3109/2000656X.2016.1151436

[19] Garusi C Manconi A Lanni G Shoulder function after breast reconstruction with the latissimus dorsi flap: a prospective cohort study—combining DASH score and objective evaluation. Breast 2016;27:78–86. 27054752 10.1016/j.breast.2016.02.017

[20] Harrington S Padua D Battaglini C Comparison of shoulder flexibility, strength, and function between breast cancer survivors and healthy participants. J Cancer Surviv 2011;5:167–74. 21225372 10.1007/s11764-010-0168-0

[21] Lee KT Mun GH A systematic review of functional donor-site morbidity after latissimus dorsi muscle transfer. Plast Reconstr Surg 2014;134:303–14. 24732650 10.1097/PRS.0000000000000365

[22] Hidding JT Beurskens CH van der Wees PJ Treatment related impairments in arm and shoulder in patients with breast cancer: a systematic review. PLoS ONE 2014;9:e96748. 24816774 10.1371/journal.pone.0096748 PMC4016041

